# Prevalence and healthcare utilization of herpes zoster and postherpetic neuralgia in South Korea: disparity among patients with different immune statuses

**DOI:** 10.4178/epih/e2014012

**Published:** 2014-08-14

**Authors:** Chelim Cheong, Tae-Jin Lee

**Affiliations:** Graduate School of Public Health, Seoul National University, Seoul, Korea

**Keywords:** Herpes zoster, Neuralgia, Postherpetic, Immunocompromised host, Prevalence, Health care costs, Korea

## Abstract

**OBJECTIVES:**

Despite the clinical and epidemiological importance of herpes zoster (HZ) and postherpetic neuralgia (PHN), their disease and economic burden related to immune status has not been studied in South Korea. Our aim was to calculate the prevalence and rate of healthcare utilization related to HZ and PHN among Korean patients stratified by immune status.

**METHODS:**

This retrospective study used the Health Insurance Review and Assessment Service National Patients Sample (HIRA K-NPS) database, which includes all medical claims from January to December 2009 on a representative sample of the Korean population. HZ and PHN patients aged ≥ 50 years were categorized into three groups by immune status: severely immunocompromised group, moderately compromised group, and non-compromised group. The prevalence, disease-related healthcare utilization, and medical costs were compared across the three groups.

**RESULTS:**

We estimated that there were 312,136 HZ patients and 48,461 PHN patients ≥ 50 years in South Korea. The prevalence of HZ and PHN was 18.54 and 2.88 per 1,000 persons, respectively, and increased with deteriorating immune status. The number of outpatient visits and hospitalization rate among HZ patients were highest in the severely immunocompromised group (4.38% and 7.52%, respectively) and lowest in the non-compromised group (3.82% and 4.08%, respectively). The average medical cost per patient in the severe group was the highest (240 US dollars) and that of the non-compromised group was the lowest (161 US dollars). No parameters were significantly different among patients with PHN by immunity.

**CONCLUSIONS:**

HZ patients with severe immunodeficiency had a higher prevalence of HZ, more outpatient visits and hospitalizations, longer hospitalizations, and higher medical costs than their counterparts did. Efforts should be made to reduce the HZ-related burden of severely immunocompromised patients.

## INTRODUCTION

Herpes zoster (HZ) is caused by reactivation of the varicella-zoster virus (VZV), which has remained latent in the ganglia and dorsal nerve roots for decades after primary infection with VZV, also known as chickenpox [1-3]. HZ is characterized by a painful unilateral vesicular rash that generally disappears within three or four weeks. The lifetime risk of developing HZ is up to 30% [3-6]. HZ results in a variety of complications, but the most common complication is postherpetic neuralgia (PHN), which is frequently described as the worst pain ever experienced [7,8]. PHN occurs in up to 19% of all HZ patients and lasts for several months to several years [4,9-15].

The incidence of HZ and PHN increases with age due to the gradual age-related decline in cell-mediated immunity against VZV and in the general immune system [1-3,8,9,16]. Similarly, a high incidence was reported among individuals with immunosuppressive conditions [2,8,9,16,17]. The economic burden on the healthcare system of treating patients with HZ and PHN also increases for older populations and among those with a compromised immune status (CIS) [2,5,6,8,9,12,17].

For HZ treatment, antiviral agents must be used within 72 hours of rash onset for maximum effectiveness [18,19]. However, timely recognition and treatment occurs in less than half of all cases, and no effective therapy for the prevention and control of PHN exists [19]. Therefore, the most effective means of reducing the burden of HZ and PHN is to prevent HZ infection through vaccination [7,20]. The live-attenuated HZ vaccine, which increases cell-mediated immunity to VZV, was approved in 2006 for use in adults from 50 or 60 years of age and older in several areas including the US, Canada, Australia, and the EU and became available in South Korea in June 2012 [21-26]. However, administration of the live-attenuated HZ vaccine can result in a disseminated disease among immunosuppressed individuals [24]. Therefore, the vaccine has been contraindicated in severely immunosuppressed individuals who are vulnerable to infection.

Before the introduction of the HZ vaccine, numerous countries have estimated the population-based disease burden of HZ from epidemiological and/or economic studies [5,6,13-15, 27-32]. While some studies excluded or were limited to immunosuppressed patients who are ineligible for the vaccine [5,13-15,27,29,31], other studies included all HZ patients regardless their immunodeficiency [6,28,30,32]. Nevertheless, little is known about the influence of immune status among HZ patients. Therefore, the objective of this study was to report (1) the annual prevalence; (2) healthcare resource utilization such as average annual number of outpatient visits, rate of emergency department visits, hospitalization rate, and prescription patterns; and (3) the annual medical costs for HZ and PHN patients with different immune statuses in South Korea.

## MATERIALS AND METHODS

### Data source

This retrospective, population-based study used the 2009 database from the Korean National Patients Sample of the Health Insurance Review and Assessment Service (HIRA K-NPS), which is a stratified database of Korean national medical reimbursement claims reviewed by the HIRA. The HIRA database contains medical claims data from all those insured under the Korean National Health Insurance (NHI) program, which is mandatory. This database contains medical information by visit, including the patient’s age, gender, reasons for each visit (represented using the International Classification Disease tenth revision [ICD-10] codes), healthcare services and medications given to the patient, and the total medical expense.

Patients in the HIRA K-NPS database were randomly selected based on 5-year age groups, gender, and whether the patient was ever hospitalized for any reason in 2009. Different sampling probabilities were applied to those hospitalized (13%) and those never hospitalized but with at least one outpatient visit (1%). All medical claims for the 711,285 patients who had been hospitalized at least once and 404,436 outpatients who had never been hospitalized were included in the database. To allow for the extrapolation of our results to the total Korean population, sampling weights were applied. All potentially sensitive patient information was de-identified.

### Study population

All patients aged 50 years and older in the database were categorized into three subgroups by their immune status: compromised immune status (CIS) group 1, CIS group 2, and non-CIS group. CIS group 1 included patients primarily diagnosed with severe CIS such as transplantation, hematological malignancies, or autoimmune deficiency disease. CIS group 2 included those who were diagnosed with mild or moderate CIS such as rheumatoid arthritis, a solid tumor, or diabetes and excluded those who were in CIS group 1. Table 1 shows details of the immunosuppressive conditions and their respective ICD-10 code. Those who were not diagnosed with any of the diseases listed in Table 1 were defined as the non-CIS group. The definitions of these conditions and code selection were confirmed based on the definitions of previous studies [5,14,15,17,27] and the opinions of clinical doctors with infectious disease specialty. HZ patients were defined as being diagnosed with HZ as the main diagnosis of their claim according to the ICD-10 codes (B02.0-B02.9). PHN patients were operationally defined as those with a main diagnosis of PHN (G53.0) among those with HZ listed as one of the five main codes at least once in 2009.

### Study measures and definitions

The prevalence of HZ and PHN was calculated in relation to the entire study population. HZ- and PHN-related healthcare utilization rate and medical costs were extracted from all medical claims with HZ and PHN as the main diagnosis. Thus, HZ- and PHN-related medical utilization and costs were analyzed separately. Information on healthcare resource utilization included the average number of outpatient visits, frequency of emergency department visits, hospitalization rate, and average length of stay among inpatients. Medical costs were collected as the amount paid out-of-pocket by the patient during visits and reimbursed by the NHI to medical providers as well as medication costs recorded in the prescription list. To estimate prescription rates according to the type of drugs, drugs on the Korean national reimbursement drug list were classified into anti-virals, non-narcotic analgesics and anti-epileptic drugs, based on the classification of the Ministry of Health and Welfare in Korea. For all estimations in this study, items not covered by the NHI were not included. All monetary values reported were inflated to the value of 2013 US dollar according to the 2013 average exchange rate that one US dollar equals 1,095 Korean won (http://data.worldbank.org/indicator/PA.NUS.FCRF) and the consumer price index adjustment factor (1.109) from the Korean Statistical Information Service (www.kosis.kr).

### Statistical analysis

Differences across the three CIS groups were compared using chi-square tests and one-way analysis of variance general linear models. All statistical analyses were performed using SAS version 9.2 (SAS Inc., Cary, NC, USA).

## RESULTS

A total of 11,502 HZ patients and 2,364 PHN patients aged 50 years and older were identified in the HIRA K-NPS database, and we estimated that there were 312,136 HZ patients and 48,461 PHN patients aged 50 years and older in the total Korean population. Table 2 shows the actual sample sizes by immune status and for those who were ever hospitalized in 2009. All data reported below were weighted.

### Prevalence of herpes zoster and postherpetic neuralgia

**Figure 1.**
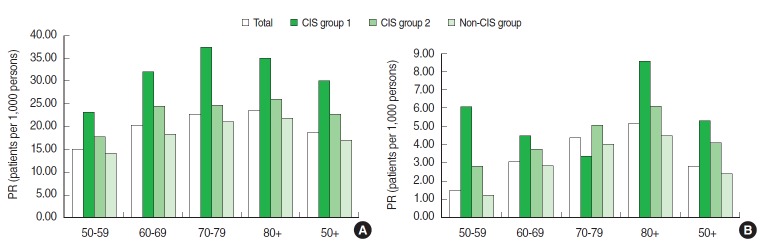
Distribution of the prevalence rate (PR) of herpes zoster (A) and postherpetic neuralgia (B) stratified by age and immune status from the 2009 Korean National Patients Sample database of the Health Insurance Review and Assessment Service in South Korea. CIS group 1 includes patients primarily diagnosed with severe CIS such as transplantation, hematological malignancies, or autoimmune deficiency disease. CIS group 2 includes those who were not in CIS group 1 and diagnosed with mild or moderate CIS such as rheumatoid arthritis, a solid tumor, or diabetes. The non-CIS group included those who were not included in CIS groups 1 or 2. CIS, compromised immune status.

The prevalence of HZ was 18.54 per 1,000 persons among our total population of those aged 50 years and older and was highest among the CIS group 1 (30.09 per 1,000 persons) and lowest among the non-CIS group (16.89 per 1,000 persons). The prevalence of HZ increased by age group from 14.72 to 23.32 per 1,000 persons for those aged 50-59 to 80 years and older, respectively. The prevalence of PHN was 2.88 per 1,000 persons in the total population and 5.32, 4.17, and 2.38 per 1,000 persons in CIS group 1, CSI group 2, and the non-CIS group, respectively. The prevalence of PHN gradually increased with age from 1.56 to 5.11 per 1,000 persons (Figure 1).

### Healthcare utilization for herpes zoster and postherpetic neuralgia management

The average number of outpatient visits due to HZ and PHN was 3.98 and 4.61 per patient, respectively (Table 3). The percentages of those who visited an emergency department or were hospitalized for HZ-related treatment in 2009 were 0.91% and 4.79% among all HZ patients, and 0.70% and 2.84% among all PHN patients, respectively. The average length of hospitalization among HZ and PHN inpatients was 10.93 and 14.75 days, respectively.

In total, 89.8% of all HZ patients received at least one prescription for HZ treatment. Most patients received antivirals or non-narcotic analgesics (67.6% or 67.9%, respectively). More than half of all PHN patients received non-narcotic analgesics or antiepileptic agents (57.5% or 53.5%, respectively). Approximately 10% of PHN patients received anti-viral agents that are known to be ineffective for the treatment of PHN.

Healthcare utilization for HZ management significantly increased with the severity of the immunodeficiency. The number of outpatient visits and hospitalization rate of HZ patients were highest in CIS group 1 (4.38% and 7.52%, respectively) and lowest in the non-CIS group (3.82% and 4.08%, respectively). However, the rate of emergency department visits and prescription use were not significantly different. Except for the prescription rate, none of the other parameters for PHN-related medical resource utilization was significantly different across the three study groups.

### Herpes zoster and postherpetic neuralgia related medical costs

The total medical expenditures related to HZ and PHN treatment in South Korea were 55.0 million US dollars and 8.2 million US dollars, respectively, according to the 2013 price adjustment for inflation. The average medical cost of HZ management was 176 US dollars per patient (Table 3).The average medical cost among CIS group 1 was the highest (240 US dollars) and that of the non-CIS group was the lowest (161 US dollars). Among those with PHN, the average expenditure for costs related to PHN management was 169 US dollars per patient. Patients in CIS group 2 incurred the highest medical costs (198 US dollars), and the non-CIS group incurred the lowest (152 US dollars).

## DISCUSSION

This study documented the prevalence and healthcare utilization patterns of HZ and PHN in South Korea by the immune status of patients using a national claims database. The prevalence of HZ and PHN was 18.54 and 2.83 per 1,000 persons aged 50 or over, respectively, and the highest prevalence rate was observed in the severely compromised group. Most measures of healthcare resource utilization for HZ management significantly increased with the severity of the immunodeficiency (*p*<0.05).

The prevalence of HZ in the total population (not stratified by age) was 10.36 per 1,000 persons (data not shown). Our calculated prevalence rate is slightly lower than that reported by Choi et al. [30] who found an increasing rate of outpatient visits among Korean patients with HZ from 7.93 per 1,000 persons in 2003 to 12.54 per 1,000 persons in 2007. This difference may have resulted from the broader definition of HZ in the study by Choi et al. [30] who included those with a PHN diagnosis in their population of HZ patients.

The prevalence reported in the present study was considerably higher than the prevalence or incidence rates of studies from other countries (range 1.74-4.97 per 1,000 persons) [1,4-6, 11-14,27,28,31,32]. This difference may have had to do with the calculation of the number of HZ patients. Previous studies reported the annual incidence of HZ; however, we calculated the prevalence of HZ using the data on patients who visited a medical facility for HZ treatment at least once in 2009. Therefore, the results of our study are likely to be higher than those of previous studies are. Ethnic differences also might contribute to the high rates reported among Asian countries. The highest incidence rate among previous studies performed outside of South Korea was 4.97 per 1,000 persons, which was reported in Taiwan [6]. Other possible sources of the disparities might be increases in HZ incidences across time due to the ageing population with a higher prevalence of immunocompromising conditions and increases in the diagnosis and treatment of HZ by the marketing of new antiviral agents such as famciclovir and valaciclovir.

The prevalence of HZ in CIS group 1 was substantially higher than that of the non-CIS group (30.09 vs. 16.89 per 1,000 persons). The high prevalence rates among the CIS groups were consistent with that of previous studies that compared the incidence rates of HZ among patients with different immune statuses [5,14,27]. In France, the incidence of HZ among the total population and those with severe CIS were 3.82 and 4.70 per 1,000 person-years, respectively [5]. In Italy, the incidence of total population and among immunocompetent people were 4.31 and 4.07 per 1,000 persons, respectively [14]. In the US, the incidence among immunocompetent people and severely compromised patients were 3.0 and 10.3 per 1,000 persons, respectively [27].

The rate of hospitalization for HZ was 4.79% among those aged 50 years or older, which is different from that in Taiwan (4.14% among those aged 60 years or older) [6] and Italy (1.3% among those aged 50 years or older) [14]. These inconsistencies might be due to differences between study designs and national healthcare settings. For example, Italy’s National Health Service model tends to use healthcare resources more strictly than Taiwan and South Korea do, both of which have social insurance system.

The majority of Korean HZ patients (67.61%) received systematic antivirals, which is consistent with the international recommendations and that reported by previous studies [12,13, 17-19,28,29]. The rate of anti-viral use in CIS group 1 was lower than that of the other two groups. Possible reasons for this discrepancy should be investigated using future prospective studies. To relieve PHN symptoms, supplementary therapy for HZ can be used, but antiviral therapy is known to be ineffective [3,18,19]. The pharmacotherapy that comprised the highest portion of PHN treatment in this study was antiepileptic agents.

The total economic burden of HZ and PHN reported in the present study was 63.2 million US dollars, which corresponded to approximately 0.25% of the total 2009 NHI costs for the Korean population aged 50 or over [33]. This economic burden reached about 37% of the medical expenditure typical for bone density and structure treatment and 72% for malignant neoplasm of the prostate among those aged 50 and older [33].

The average medical costs of HZ and PHN management per patient were 176 and 169 US dollars, respectively, in this study. In previous studies from South Korea [30], the US [15,17,29], the UK [12,13], Italy [14], and Spain [32], the average medical costs per patient were approximately 200, 431-1,112, 165-317, 231, and 517 US dollars, respectively (assuming 1 euro and 1 British pound equals 1.39 US dollars and 1.60 US dollars, respectively, http://www.fxcentre.com/AverageRates-2011.pdf).

Costs per HZ patient were higher among those with more severe immunosuppression, ranging from 161 US dollars among the non-CIS group to 240 US dollars among CIS group 1 (*p*< 0.05). Previous studies in the US reported the average cost per HZ patient to be almost two to three folds higher among patients with severe CIS than that among their counterparts [15, 17,29]. However, neither the prevalence nor healthcare utilization among PHN patients significantly differed across immune status in our study. These unexpected results might be related to the small sample size of each PHN subgroup (Table 2), the strict definition of PHN patients (PHN had to have been the main reason for the visit), and/or data spanning over only one year in our study. For an appropriate sample size, at least 20 to 30 subjects per group are needed to reveal any significant differences [34]. In addition, PHN patients are required to receive treatment for longer than one year; therefore, the medical costs calculated over our study period include only a part of the entire treatment schedule. Prospective studies are required to further understand the burden related to PHN.

Further efforts to reduce the burden of HZ should be focused on the immunosuppressed population who are more susceptible to HZ infection and have higher utilization of healthcare resources than the non-CIS population do. The severely immunosuppressed population is ineligible for the HZ vaccination, which is the most effective method of preventing the infection. Moreover, the prevalence of HZ infection and the magnitude of burden are expected to increase in aging populations.

A major strength of this study was the use of complete medical claims data from a representative sample of the entire population in South Korea, which included demographic data, diagnostic codes, prescription lists, and utilization records of all related healthcare resources covered by the NHI. However, these administrative data has the following limitations. First, consistent with other population-based studies on disease burden, the calculated prevalence in the present study was based on retrospective data. Therefore, preexisting illnesses or comorbidities may have been incorrectly coded in the database, thus would have been incorrectly used to define HZ and PHN patients and their immune statuses. For example, a substantial number of HZ and PHN patients received no medication during the study period (10.17% and 12.76%, respectively). Second, items not covered by the NHI were not included in our estimation of medical costs. According to the 2009 National Health Insurance Service report in South Korea, the average out-of-pocket payment for items not covered by the NHI was approximately 13% of a patient’s total medical costs [35]. Therefore, the medical costs reported in the present study seem to be an underestimation of the actual costs. Third, we extracted patients’ medication information from the prescription list included in the medical claims data. The actual amount of medication dispensed to patients may have been smaller than that estimated by the present study. Moreover, dispensing fees were not included. Finally, our stratification by immune status was based on retrospective diagnostic information in the claims database. Further research on HZ and PHN patients should use actual test results to determine immune status.

To our knowledge, this was the first study to estimate the prevalence and the healthcare utilization patterns of HZ and PHN stratified by patient immune status from a national database in South Korea. With the recent trends of a rapidly aging population and increasing prevalence of immunosuppressive diseases, the burden of HZ will likely increase in South Korea. Efforts should be made to prevent HZ and reduce its burden among the severely immunocompromised patients who remain susceptible to HZ infection and PHN.

## Figures and Tables

**Table 1.** ICD-10 codes used to define the severity of patients’ immune statuses

Immune status	Conditions	Codes
Severe CIS	Transplantation (hematopoietic stem cell, solid organ)	Z94.0-Z94.4, Z94.8-Z94.9
	Certain hematological malignancies (Hodgkin’s lymphoma, multiple myeloma, acute leukemia, non-Hodgkin’s lymphoma)	C81-C96, D00-D09, D37-D48
	AIDS, advanced HIV infection, and other hematological diseases (aplastic anemia, agranulocytosis, myelodysplastic syndrome)	B20-B24, D46, D61, D70
	Other disorders involving immunodeficiency	D80-D84, D89
Mild or moderate CIS	Immune-mediated diseases (rheumatoid arthritis, systemic lupus erythematosus, inflammatory bowel disease, Wegener’s granulomatosis)	M05, M06, M08, M09, M31.3, M32, M32.1+, M32.8, M32.9, K50, K51
	Cancer (solid tumor)	C00-C80, C97
	Diabetes	E10-14
	Renal insufficiency (chronic renal failure, hemodialysis)	N18.0, N18.8, N18.9, N19
	Hepatic insufficiency (liver cirrhosis)	K74.0-K74.6

ICD-10, International Classification of Diseases tenth revision; CIS, compromised immune status; AIDS, acquired immune deficiency syndrome; HIV, human immunodeficiency virus.

**Table 2.** The actual sample size for each immune status group

	CIS group 1^1^	CIS group 2^2^	Non-CIS group^3^	Total
HZ	509	4,760	6,233	11,502
Inpatients^4^	34	654	1,735	2,423
Outpatients^4^	475	4,106	4,498	9,079
PHN	90	1,119	1,155	2,364
Inpatients^4^	6	100	222	328
Outpatients^4^	84	1,019	933	2,036

HZ, herpes zoster; CIS, compromised immune status; PHN, postherpetic neuralgia.

1 Includes patients primarily diagnosed with severe CIS such as transplantation, hematological malignancies, or autoimmune deficiency disease.

2 Includes those who were not in CIS group 1 and diagnosed with mild or moderate CIS such as rheumatoid arthritis, a solid tumor, or diabetes.

3 Includes those who were not included in CIS groups 1 or 2.

4 In this stratified sample of the Korean population, a different sampling probability was applied to those who were hospitalized (13%) and those never hospitalized but who visited an outpatient clinic at least once (1%).

**Table 3.** Mean herpes zoster (HZ)- and postherpetic neuralgia (PHN)-related healthcare utilization per patient

	HZ				PHN			
	CIS group 1	CIS group 2	Non-CIS group	Total	CIS group 1	CIS group 2	Non-CIS group	Total
Population (n)	7,054	96,984	208,098	312,136	1,246	17,838	29,377	48,461
No. of outpatient visits	4.38	4.30	3.82	3.98^1,*^	3.46	4.97	4.43	4.61
Rate of ED use (%)^2^	1.20	1.02	0.85	0.91	0.00	1.08	0.50	0.70
Hospitalization rate (%)	7.52	6.12	4.08	4.79^*^	4.94	3.15	2.57	2.84
Length of hospitalization (d)	11.12	10.85	10.97	10.93^3,*^	9.50	15.25	14.81	14.75
Medications (%)	86.8	90.6	89.6	89.8	95.7	89.8	85.3	87.2^*^
Anti-virals	59.8	68.3	67.6	67.6	4.9	9.1	9.9	9.5
Non-narcotics	61.1	69.1	67.6	67.9	72.2	58.5	56.3	57.5
Anti-epileptics	22.0	24.3	19.8	21.3^*^	58.6	51.8	54.3	53.5
Medical cost (US dollars)^4^	240	205	161	176^*^	166	198	152	169^*^

All figures were weighted for extrapolation to the general Korean population in 2009.

CIS, compromised immune status; ED, emergency department.

1 Calculated by dividing the total number of outpatient visits (1,436,352 visits) by the number of all HZ patients (383,128).

2 The percentage of those with any history of an emergency department visit during 2009 among all patients.

3 Calculated as the sum of inpatient days related to HZ (177,376 days) divided by the number of all HZ inpatients (16,538).

4 Costs are presented as 1 US dollar equals 1,095 Korean won according to data from 2013.

* p<0.05.
